# Getting To(wards) Know(ing) Together: An Innovative Collaborative Approach in Residential Care for People with (Severe) Intellectual Disabilities and Behaviour That Challenges

**DOI:** 10.3390/ijerph22091368

**Published:** 2025-08-30

**Authors:** Gustaaf F. Bos, Vanessa C. Olivier-Pijpers, Alistair R. Niemeijer

**Affiliations:** 1 Department of Care Ethics, University of Humanistic Studies, Postbus 797, 3500 AT Utrecht, The Netherlands; 2 Care Organization Ipse de Bruggen, Postbus 7027, 2701 AA Zoetermeer, The Netherlands

**Keywords:** intellectual disabilities, challenging behaviour, highly specialized residential care, socialisation, insider-outsider, stagnant care practices, complexity, collaborative research, community of practice

## Abstract

People with moderate to severe intellectual disabilities (M/S ID) and behaviour that challenges are still almost exclusively encountered and understood within a highly specialized professional care system context. They are almost invisible in the societal mainstream, where a wider variety of perspectives on (everyday) manners, encounters, relationships and life applies. These (and other) exclusionary dynamics render everyday relations with residents with M/S ID whose behaviours challenge still largely dependent on the interpretative frameworks and actions of professionals. Professionals are trained and socialized within highly specialized professional care system contexts, despite a growing scientific and professional awareness that behaviour that challenges is a multifaceted and contextual phenomenon. In this paper, we report on a pioneering initiative (titled Project WAVE) which aimed to cultivate a fresh and comprehensive approach to behaviours that challenge within stagnant care practices. Our goal was to foster an innovative collaborative paradigm by facilitating an extensive and enduring exchange between “insiders”—professionals of specialized care system contexts—and “outsider-researchers”—individuals socialized through alternative avenues. We present our epistemological and methodological approach, the data collection process (a multiple case-informed community of practice), and the most important lessons learned.

## 1. Introduction

In the Netherlands, contrary to three decades of international deinstitutionalization movement [1], people with moderate to severe intellectual disabilities (M/S ID) and behaviour that challenges [2] are still almost exclusively encountered and understood within a *highly specialized professional care system* context. They are almost invisible in the societal mainstream, where a wider variety of perspectives on (everyday) manners, encounters, relationships, and life applies [3]. Hence, in the unlikely event that people with M/S ID and behaviour that challenges become visible to mainstream society (e.g., in a documentary or a news-item) they are primarily framed as clients, patients, or confused people, rather than fellow human beings (with relatable feelings, interests, ways of life, etc.). Furthermore, most members of the general public—who are unfamiliar with the logic, language, structures, and norms of a highly specialized professional care system context (e.g., casual passersby or neighbours without ID)—tend to assume that only care professionals will know best ‘how to deal with them’ [3].

These exclusionary dynamics render everyday relations with a resident with M/S ID whose behaviours challenge still largely dependent on the interpretative frameworks and actions of professionals, who are trained and socialized within those highly specialized professional care system contexts, despite a growing scientific and professional awareness that behaviour that challenges is a multifaceted and contextual phenomenon [2,4,5]. Highly specialized professional care system perspectives become even more dominant by the fact that most residents with M/S ID cannot speak (for themselves), and often lack personal relationships (even with their relatives); their social networks mainly consist of paid staff [6,7,8].

Since everyday life in highly specialized residential care settings for people with M/S ID and behaviours that challenge tends to be complex and disruptive for everyone involved, staff often resort to a regime based on values like tranquillity, regularity, safety, and risk prevention [3,8]. These primary staff values are operationalized in a wide variety of ways, ranging from supporting ‘clients’ mainly via distinct approaches [9,10] and applying highly-structured and/or low-stimulant living arrangements [11,12] to confining ‘difficult clients’ for extensive periods of time [13]. Accordingly, the urge to stay calm, predictable, and risk averse extends from the remote location and functional design of residential homes to a rather strict interpretation and enactment of comprehensive day programs and protocols, as well as to prevailing views on good professionalism and on what counts as relevant knowledge in education and practice [14]. Furthermore, the health inspectorate and increased media attention for incidents and concomitant bad practices, mirror the ongoing societal, governmental, and legislative pressures to prevent harm and avoid risk [15,16,17].

Unfortunately, the pervasive *risk reduction focus* also tends to fuel a rather distant and emotionally neutral way in which (future) professionals are trained and socialized to relate to people whose behaviours challenge, yielding little interpersonal connection [18,19]. And if—despite the risk reduction focus—scarce interpersonal relationships do emerge between ‘clients’ and ‘staff’, they tend to be volatile and precarious, since complex care settings are characterized by high staff turnover [5], which in turn is considered a trigger for challenging behaviour [20,21]. Consequently, the depersonalized and decontextualized logic of risk prevention that ensues is enacted by professionals-under-pressure, in continuously changing team compositions, who tend to face their often socially isolated clients in an asymmetrical and potentially othering relationship [19,22].

The failure to incorporate a wider variety of perspectives and knowledge sources when addressing behaviour that challenges parallels the poignant analogy presented by novelist Ngozi Adichie, known as the ‘danger of a single story’ [23,24]. This concept elucidates the peril of relying solely on default and incomplete assumptions, conclusions, and decisions, thereby enabling inadequate framing and misunderstandings. Adopting a singular narrative lens toward challenging behaviour inhibits the exploration of potentially enriching, multifaceted insights into entrenched, or even stagnant, care practices. Such an approach appears discordant with Clifford Simplican’s impassioned plea to honour the intricate complexity inherent in said behaviour cf. [2,25].

To our knowledge, empirical studies that try to account for this complexity are currently lacking. Hence, as scholars in the field of disability care, we embarked on a pioneering initiative (titled *Project WAVE*) to cultivate a fresh and comprehensive approach to behaviours that challenge within stagnant care practices, by explicitly making space for alternative perspectives and knowledge sources. We did so by drawing on the conception of collaborative research as ‘an *epistemic partnership* that neither necessarily starts from a shared (research) question nor aims at realizing a common political goal or shared results, but rather engages in common, systematic, and reflective efforts to expand forms of knowledge and practices of [everyone] involved’ [26]. The name Project WAVE was chosen to symbolize our aspiration to create movement and drive change—much like a wave begins with a small shift and expands across a surface. To fuel this innovative paradigm, we assigned “outsider-researchers” (individuals without care training or background, who were socialized through alternative avenues) [27] to the stagnant care practices, to collaborate for a prolonged period with the “protagonists” (persons with M/S ID), “insiders” (professionals who are trained and socialized within specialized care system contexts), and relatives. Our guiding research question was: *Which appropriate and sustainable knowledge does a long-term collaboration between outsider-researchers, protagonists, insiders, and relatives produce?* By facilitating an extensive and enduring exchange between these outsider-researchers, protagonists, insiders, and relatives, we hoped to:

(1) contribute to the quality of care and life for the protagonists, their relatives, and care staff, and
(2) gain valuable insights for stagnant care practices elsewhere, as well as for the education of (future) care professionals.

In this paper, we present our methodological approach, the epistemological notion of *getting tow(ards) know(ing) together*, the data collection process, and the most important lessons about this approach and process. Through this, we primarily aim to highlight both what it requires and the great potential it holds to structurally create space for multiple perspectives in research in residential care settings for people whose behaviours challenge. For an in-depth description and analysis of the project outcomes, see Bos et al. [28].

## 2. Methodological Approach

### 2.1. Selection of Cases and Outsider-Researchers

Project WAVE (2019–2022) revolved around 12 cases, each consisting of one person with M/S ID (hereafter: protagonists), their care staff (hereafter: insiders), and relatives, as well as the patterns, systems and physical contexts of the stagnant care practices in which they interacted daily, and within the larger context of the care sector. According to Olivier-Pijpers et al., stagnant care practices are characterized by a large and frequently changing team of professionals surrounding the client, strained relationships between professionals and relatives, the seemingly insurmountable task of managing challenging behaviour, and a persistent threat of incidents [29]. Given the challenging nature of our project rationale and the unpredictability of its impact, it was essential to work with partners who, above all, trusted us. Therefore, our case selection was based on convenience sampling via pre-established networks of the research team. The main criterion for she stagnant care practices in our selected cases was that they persisted despite ongoing extra care budgets and/or frequent expert consultation, provided by the government sponsored national Centre for Consultation and Expertise (CCE)—which is generally considered a last resort. Therefore, there was a broadly shared need for new insights and approaches that would help facilitate the continuously stagnant care practices into a more dynamic and sustainable mode for everyone involved. We selected 12 cases within 6 different residential care organisations (two in each), in close collaboration with the responsible behavioural specialist and manager. Each case concerned a sheltered residential facility accommodating between 4 and 7 residents.

Following case selection, each case was assigned an outsider-researcher for approximately two years, with a weekly commitment of around four hours. In order to select outsider-researchers, we had designed a process aiming to identify and score the following traits: *good heartedness, sensitivity, perseverance, stubbornness, reflexiveness, non-intrusiveness,* and *ability to collaborate*. As part of this process, three conversations were held and participants reflected on a filmic portrait of a person with challenging behaviour, his caring staff, and relatives. As a final selection criterion, we scored the *distinctive and complementary nature* of each person’s perspective. This resulted in a group of 12 self-aware and socially sensitive people, with potentially relevant professional and/or personal backgrounds (other than in healthcare) which strongly influenced their sense of identity, view of man, and/or approach to life. Once selected, the outsider-researchers were primarily allocated to a case on the basis of travel distance; after all, they needed to be able to sustain their relatively small weekly time commitment over two years.

### 2.2. Outsider-Researchers and How They Present Themselves, Protagonists and Insiders

#### 2.2.1. Anne, Rian, and Insiders

Anne works as a social designer, and considers herself a curious and creative person. About her first visit to protagonist Rian, Anne says: ‘The first time I was in the house, I felt like I was in the way. As if I hadn’t come at the right moment. You can tell that Rian is physically strong, and at first, I found that a bit intimidating. At the same time, I was fascinated by the observations she shared with me. Like when we took a short walk outside and talked about how the place felt like a regular neighbourhood. Rian said: “But there are a lot of wheelchairs here”.’ After a year, Anne shares with and about the insiders what stood out for her: ‘Many support staff don’t know how to spend meaningful time with Rian or how to build a relationship of trust with her. The music therapist, however, has been visiting Rian for years and is always welcome—no matter how she’s feeling. How do those two manage that?’ After two years of involvement, by the end of her assignment, Anne describes Rian as follows: ‘Rian is curious and attentive. She told me, for example, to drive carefully when leaving her place, because I had once mentioned I got a speeding ticket. She’s excessive and always wants more—she’ll try to keep you with her as long as possible. And if there are three cream puffs on the table, it wouldn’t occur to her that one might be for someone else. She loves to joke around. When I come in and ask her: “Well? Do you still remember me?” she flashes a big grin and says: “Noooo”.’

#### 2.2.2. Annemiek, Elisa, and Insiders

Annemiek is a (foster) mother and a journalist. She describes herself as curious, reliable, and involved. About her first visit to protagonist Elisa, Annemiek says: ‘It’s a beautiful day when I first meet my main participant, 23-year-old Elisa, in April 2019. She’s at the petting zoo with her day program group, gazing blissfully at the sky, her face turned toward the sun. Now and then, she stretches her arms and back in what seems to be excitement and pure pleasure. I see a joyful young woman who clearly loves warmth.’ After a year, Annemiek shares with and about the insiders what stood out for her: ‘Exploratory questions. Are you allowed to comfort Elisa when she’s sad, or sit with her when she’s too afraid to fall asleep? And can too much calm also be undesirable? I would have liked to continue weaving together insights with the insiders and her family through these questions—but unfortunately, in January 2020, I was summarily dismissed by the care organization.’ After a year of involvement, Annemiek describes Elisa as follows: ‘At the day program, Elisa often sits in the hallway in front of another group’s room, where people are shouting loudly. When there are moving images or music playing on my laptop screen, she’s right up close to it. I often see signs of her curiosity. Staff tell me that she goes out with her mother—whom I unfortunately never met or spoke to—to places like the snack bar, a fairground, or a festival. So, she can handle stimulation. At the facility and during day activities, she can spend hours doing very little—just moving around a bit, gazing at the ceiling—and she looks content. This suggests that finding a balance between calm and stimulation is important, and that we shouldn’t avoid offering her activities or making small demands out of fear that Elisa might become overwhelmed.’

#### 2.2.3. Boris, Ronald, and Insiders

Boris is father, a 2D character animator, animation teacher, gardener, and avid rower. He describes himself as sensitive and intuitive. About his first visit to protagonist Ronald, Boris says: ‘To be honest, I found it difficult to get clear on what exactly I was there to do. Because I wasn’t sure myself, I think I came across as uncertain to the support staff as well. I had seen a documentary featuring someone with challenging behaviour and had mentally prepared for the worst—things like self-injury and being non-verbal. But when I met Ronald, none of that turned out to be the case. I was actually quite relieved. Ronald turned out to be very articulate. Later, I would come to realize that this was also a pitfall for me—precisely because, as his support staff put it, he is able to mask his disability quite well through his verbal abilities.’ After a year, Boris shares with and about the insiders what stood out for him: ‘The team appears to place strong emphasis on the factual accuracy of Ronald’s statements. They come across as highly focused and professional. But do they ever question their interpretations?’ Boris went on to introduce questions of wonder in his interactions in the case. ‘Does it all have to be true? I wanted to experiment together with the power of imagination and beauty’. After two years, Boris describes Ronald as follows: ‘Ronald is a kind soul. A sensitive man with his heart in the right place. He loves good food. And under the right circumstances, he can come out with surprisingly beautiful statements. Like: “Every day you wake up, you should be grateful.” Ronald is a good storyteller. Someone who’s been through a lot in the past, which makes him more appreciative of his current situation. He can get nostalgic when talking about his foster parents. I also found him surprisingly honest when he spoke about the grief he felt after the death of his foster father.’

#### 2.2.4. Jan, Mark, and Insiders

Jan works as a Salvation Army soldier. He has lived experience in societal margins and describes himself as persistent, trustworthy, and a good listener. About his first visit to protagonist Mark, Jan says: ‘I was very nervous and had no idea what to expect; when I first saw Mark, I just let everything wash over me. I found the term ‘challenging behaviour’ quite frightening—it could mean anything: aggression, or behaviour that’s hard to understand, for example.’ After a year, Jan shared with and about the insiders what stood out for him: ‘There is a constant turnover of support staff in Mark’s life. These people, who only work with him for short periods, mostly hear that he is aggressive. Both factors contribute to Mark struggling to build a positive relationship with almost anyone. How can the care team contribute to greater joy in life for Mark and his housemates, as well as for those who support him? For example, by encouraging the organization to adopt a more friendly and relational approach to caregiving.’ After two years, Jan describes Mark as follows: ‘Mark is really like a little child who needs a lot of attention. He loves jokes and singing. Lego is also one of his favourite activities. He’s very fond of his family and sometimes gives out little kisses. He really enjoys swimming. I found it very difficult to connect with him at first, but through small steps I was able to build a kind of friendship with him. I also noticed that he can sometimes be unpredictable. At times, he insists on getting his way, just like a small child might. Mark really enjoys going for walks. I think Mark is searching for stability, warmth, love, and a sense of security. In my view, it has been very difficult for him to find that during the time I’ve been involved with him. We’ve gone through around 40 support staff, three behavioural specialists, and three managers. And the turnover just keeps going. I believe this makes Mark feel very unsettled and insecure. This is not a solid foundation for building a stable life.’

#### 2.2.5. Marcel, Özcan, and Insiders

Marcel is a father, a former soldier in the Dutch Special Forces, and a former trail runner. He describes himself as a life lover and enthusiast. About his first visit to protagonist Özcan, Marcel says: ‘I was quite nervous the first time I made my way to the place where Özcan lived and received care. What would the location look like? What kind of clients lived there, and what did the support staff look like? And most of all: how would Özcan respond to me—and how would I respond to him? These were the questions going through my mind on the way there.’ After a year, Marcel shares with and about the insiders what stood out for him: ‘Everyone works very hard, but it’s unclear from which vision, within what collaborative structure, and with what responsibilities. What I contributed afterward was mainly: Improving teamwork: who is responsible for what, and for what purpose?’ After a year and a half of involvement, Marcel describes Özcan as follows: ‘Özcan is, at heart, a golden guy with a good soul. There’s not a trace of malice in him—and that’s how he views the world around him, too. His passion for fireworks sometimes gets in the way, because he lacks the cognitive ability to distinguish seasons and the appropriate context for things like that. He loves music—Turkish music!—and enjoys a good joke now and then. I’ve never known him to be anything other than a cheerful guy.’

Marcel left his role as an outsider-researcher earlier than planned, as a new job demanded his time and attention. Catrien then took over his position as a new outsider-researcher.

#### 2.2.6. Catrien, Özcan, and Insiders

Catrien works as an investigative journalist. About her first visit to protagonist Özcan, she says: ‘It was a meeting like so many others. I was introduced to Özcan. I reached out my hand, introduced myself, he shook my hand and told me his name. From his speech, I could tell he might be a little ‘different’, but that was it. What made it feel different was the setting: we were in a ‘living room’ that didn’t feel like a real living room. There were support staff around, keeping an eye on him. If I had met him on the street, it would have been just like any other introduction. I smiled at him as we shook hands, and I think he smiled back. A support worker directed me to a chair and told me to wait. I wasn’t supposed to speak to Özcan. He was putting plates into the dishwasher because he had been asked to do so. As he went about his task, he kept glancing back at me. I smiled at him. He smiled back, then quickly turned his head away. And soon after, he looked again. It seemed like he was curious and trying to make contact. That’s what I wanted too—to make contact. There was nothing ‘hard to reach’ about him to me.’ After a year, Catrien reflects: ‘I would have liked to get to know Özcan, but I was only allowed to meet him once—if you can even call it an encounter. I was allowed to watch him, but I was told it was better not to ask him questions or speak to him. Everything I ‘know’ about him comes from the staff. They told me he loves fireworks, but that he’s also afraid of them. They told me he sometimes gives people spontaneous hugs, and that he likes to wear gel in his hair and cares about nice clothes. They told me his mother is very important to him—every day he counts down the days until he gets to go home to her, one night a week. He himself only told me that he is Turkish. He struck me as gentle. What I do know is this: once, he wasn’t a man living in a care facility, but a little boy at home with his family. Once, he wasn’t someone an unfamiliar adult wasn’t supposed to talk to, but a child sitting in a primary school classroom. Who that boy was—maybe only his mother truly knows. I would have loved to speak with her, but I wasn’t allowed to.’

#### 2.2.7. Mark, Casper, and Insiders

Mark is an advisor and coach for organizations and teams. He describes himself as passionately curious, service-oriented, headstrong, and loving. About his first visit to protagonist Casper, Mark says: ‘I first met Casper in the hallway of the facility where he lives. He’s on his way to the gym. His father is with him. The first contact is one-sided: I observe him with curiosity. Casper is a slim, well-built, agile young man. Blond, blue-eyed, spiky hair, slightly prominent front teeth, and an intense gaze. Casper walks right past me without a glance. He also walks right past his father, who takes his hand and turns him toward him. Casper pays no attention to me. Not at all. Oof, that’s uncomfortable! How am I supposed to build something with him if he doesn’t even seem to notice me?’ After a year, Mark shared with and about the insiders what stood out for him: “The care team is extremely professional and competent. They are always busy (‘doing time’ and ‘care time’), hardly allowing themselves any time to relax or reflect. What I mainly contributed afterward was: The importance of ‘time-to-be’ and thematic learning.’ After two years of involvement, Mark describes Casper as follows: ‘Casper is always the same, and always different. He is always strong, solid, and fast. Focused and intense. He often seems completely absorbed in himself, yet at times, it’s as if he suddenly loses that sense of self. He evokes tenderness in me. And sometimes—when he’s in one of his grabbing, squeezing moods—I feel a little afraid of him. Then I’m cautious and alert. Very occasionally, he gives a deliberate response to a touch, a toy, or a laugh. But most of the time, he doesn’t. He is changeable, like a leaf on a tree in autumn: constantly moving in the wind, pulled in all directions. It doesn’t know when a gust will come, or from which direction. With Casper, the wind seems to blow from within. And neither of us ever knows from which way it will come. When Casper and I are together, we are together. We take our time—time to be.’

#### 2.2.8. Mats, Henk and Insiders

Mats works a social designer. He describes himself as outspoken. About his first visit to protagonist Henk, Mats tells: ‘In my world, I had never encountered people with challenging behaviour before. Because of that, I didn’t know what to expect. At my first appointment at Henk’s care facility, I was sitting in a small office with the behavioural specialist and the residence coordinator. One horror story after another was told to me about Henk’s aggressive outbursts. Until the case manager suggested I go and meet Henk. I was super nervous as I walked down the hall to his room. It was the only locked room. The only room the on-duty staff had to accompany me to, just in case. It felt like we were walking toward a cage holding a hungry, unpredictable tiger. I felt out of control. I had no idea what to expect or what to do if something happened. But nothing happened. Inside the room was an old man in pyjamas who kindly shook my hand and then asked the caregiver if he could keep me there.’ After a year, Mats shared with and about the insiders what stood out for him: ‘The care team seems so focused on preventing incidents that Henk’s life has taken on a form and rhythm none of us would wish for ourselves: grey, monotonous, and without anything to look forward to. What I mainly contributed afterward was stressing and experimenting the importance of fostering moments of happiness in everyone’s life; while also accepting and responding to the occasional hangover—in Henk’s life a bad day marked by more challenging behaviour.’ After two years, Mats describes Henk as follows: ‘Henk is a sweet child trapped in an old body. He wants nothing more than to do right by you, and he quickly feels guilty if he thinks he has disappointed someone. There’s no bigger fan of Disney than he is. Pink is his favourite colour. He wants to belong, but the world moves too fast and is too intense for him to keep up.’

#### 2.2.9. Peter, Raymond, and Insiders

Peter is a former police officer and a resident of a four-generation home. He describes himself as imaginative. About his first visit to protagonist Raymond, Peter says: ‘It was a drive of more than an hour. I had serious doubts about whether I could truly contribute to the circumstances Raymond was living in. Still, the idea of being an outsider in an unfamiliar professional environment was appealing. What we would call ‘understandable behaviour’ is already hard to interpret.’ After a year, Peter shared with and about the insiders what stood out for him: ‘The care team learns in a catastrophic way because they do not learn from and with each other, but instead repeatedly consult their manager as a sort of Judge Judy to decide what should be done. What I mainly contributed afterward was: encouraging and coaching the individual team members, as well as the team as a whole, to (re)engage in learning together about daily caregiving practices.’ After two years of involvement, Peter describes Raymond as follows: ‘Raymond is a child in an adult’s body. Caged when indoors, strong and physical when outdoors. He loves being outside, enjoys nature and animals. He lights up when he sees familiar people, especially family members. He’s afraid of changes and reserved around fellow residents and strangers. He clings to daily routines, chores, and rituals. He has a high pain threshold and rarely indicates when something is wrong. He speaks in short, fairly intelligible, simple sentences. He flashes a broad smile when he recognizes you. He enjoys children’s programs and cartoons.’

#### 2.2.10. Sanneke, Sandra, and Insiders

Sanneke is mother and social designer. She describes herself as empathetic, having a strong sense of justice, artistic and analytical. About her first visit to protagonist Sandra, Sanneke tells: ‘I was nervous about meeting my protagonist Sandra. How would her hard-to-understand behaviour affect me? What kind of person would she be? I had been informed beforehand that Sandra would not actually meet me: she is blind. I was asked to observe quietly and invisibly, from her perspective. I found this confusing, because someone who is blind must have very sensitive other senses, right? That morning, I carefully chose my clothes and shoes to make as little noise or distraction as possible. I also bought some tasty cookies for Sandra and her caregiver. The actual ‘meeting’ was shocking—not because of Sandra or her expression, but because of the unrest and confusion caused by her surroundings. During that first hour, things happened like: a caregiver saying dehumanizing things about Sandra within her hearing; caregivers standing around Sandra discussing how she should be restrained—as if she wasn’t there.’ After a year, Sanneke shared with and about the insiders what stood out for her: ‘There is great variation in how Sandra is treated. Her caregivers don’t seem to be aware of each other’s approaches; there is little cohesion in the care team, with high turnover and many temporary workers. Many team members feel unsupported by other layers within the organization.’ She mainly contributed afterward: ‘Together, together, together. The importance of interpersonal connection and caring for each other as the foundation for good care.’ After two years, Sanneke describes Sandra as follows: ‘Sandra loves music, singing along, and dancing. She enjoys good food, exploring different flavours and textures. She appreciates it when you let her know you’re there and that you care for her. She explores the world through touch and feeling. She loves speed—being in the car, speeding through curves, and going to fairs. Sandra is up for fun and celebration. She is someone who needs love and wants to give love.’

#### 2.2.11. Nout, Henk and Insiders

Nout is a father, drummer, and drum teacher. He describes himself as receptive. About his first visit to protagonist Henk, Nout tells: ‘I was nervous but also really looking forward to it. The place smelled a bit strange. Kind caregivers; I sensed years of experience. Respect. It was sunny. Henk was lying in one of those ‘boxes.’ As soon as I came in, I thought: “This must be him.” Mostly on his back, making lots of noises and playing with a kind of plastic ‘rattle.’ He was very restless. After a while, a caregiver opened the little door and took off his denim jacket, because it was warm. He dropped it on the floor. She said to him, “Hey, pick that up”’ and he immediately picked it up for her. He can’t speak, but I think he understands a lot. Regarding so-called hard-to-understand behaviour; at first, I was more cautious because I didn’t know if Henk might get very angry. But that’s not the case.’ After a year, Nout shared with and about the insiders what stood out for him: ‘The care team’s time and attention are strongly focused on the physical care of Henk and his housemates. Additionally, it is difficult to maintain Henk’s attention for extended periods. For these two reasons, the caregivers find it challenging to engage in activities with him. What I primarily contributed afterward, was that with music, you can create a “little atmosphere tent” in the living room, in which everyone—including Henk—can participate in their own way. Like you can also do at home, with your children, with a few blankets and a table for example.’ After two years. Nout describes Henk as follows: ‘Henk is a very inwardly isolated man, yet at the same time highly sensitive to his environment. That is what I find both intriguing and tragic about him. No matter how difficult it is to read his situation, he has a sense of humour and mischief. One day, I told Henk that I was feeling sad and would like a hug. He stood up calmly and resolutely and embraced me. That is also who Henk is. Due to his autistic traits, he desires a lot of structure but also frequently experiences under-stimulation.’

#### 2.2.12. Titus, Tamara, and Insiders

Titus is a husband, father of two daughters, and youth coach of the Dutch Table Tennis Association. About his first visit to protagonist Tamara, Titus says: ‘I arrived just before breakfast. The lead caregiver at the time welcomed me, and later Tamara joined for breakfast. I found Tamara to be quite ‘normal’. I watched the environment with interest—residents moving about in all directions and the organized chaos unfolding around them. Later, I accompanied Tamara to her day program. I only encountered the hard-to-understand behaviour much later. For a long time, I had no idea what might happen during an escalation or an episode of self-harm. At first, I actually couldn’t imagine Tamara ever doing such things. I really enjoyed entering this new world. I was surprised by how quickly I felt at home and adjusted to the environment. At the day program, I did feel a little uncertain at first, because the residents’ behaviour can be unpredictable—but that feeling quickly passed.’ After a year, Titus shared with and about the insiders what stood out for him: ‘The team supporting Tamara is a close-knit group, not easily open to outsiders. Their contact with other layers of the organization is strained; however, they are very attuned to what Tamara needs.’ What he primarily contributed afterward: ‘I promoted body awareness and movement. And shared my tendency to romanticize the movements between people—like when Tamara started working with a movement therapist [Dutch: bewegingsagoog]: ‘May I have this dance?’ After two years, Titus describes Tamara as follows: ‘Tamara is a girlish young woman. She often acts playfully, makes jokes or teasing remarks. She seems to seek a lot of affirmation from those around her. She is verbally gifted—she speaks well and enjoys doing so. She frequently initiates contact through conversation and questions. She has a particular fondness for dates, like birthdays. Tamara carries deep sadness. She has been hurt in the past, though this is never spoken about openly. She tends to be suspicious, but once she begins to trust someone, she can go very far in that trust. In her sessions with movement therapist Falko, she follows him blindly. She enjoys having a task, having a sense of purpose. She loves listening to music, especially K3 (a popular children’s girl group from the Netherlands and Belgium).’

### 2.3. Preparation and Training of Outsider-Researchers

The preparation and initial familiarization of the outsider-researchers involved a high degree of on-the-job training. To avoid steering their perspective and approach too much beforehand—and to make use of their *outsider* position—they received little substantive instruction. Instead, they were primarily asked to get acquainted with the protagonist and other individuals involved in the case. They were also requested to report on each visit to the principal investigator in a manner that suited them personally. These reports varied between written summaries, visual reports, voice messages, videos, etc., to which the principal investigator responded with open and in-depth questions aimed at gaining deeper insight into each person’s perspective. In this way, a steady stream of reports, emails, phone calls, and messages was established. The principal investigator also served as the primary point of contact for methodological questions, as well as a sparring partner and/or coach. Initially, contact with most outsider-researchers was weekly, but this became less frequent over time. Additionally, six quarterly meetings were organized for the outsider-researchers. These meetings were partly intended for methodological and practical support, partly for dialogical analysis, partly for exchange, and partly for peer support.

### 2.4. Design: A Multiple Case-Informed Community of Practice

In this long-term collaboration between in- and outsiders in each case we had to remain critically aware of the assimilative forces of socialisation shaped by the highly specialized professional care system logic (see Section 1), which could easily override the initially ‘fresh’ perspectives of outsider-researchers [3,30,31]. In order to facilitate an ongoing and sustainable exchange between outsiders’ perspectives and insiders’ expertise, we embedded the cases within a *community of practice* [32]. A community of practice is a group of people who share a common interest or passion for a specific field, and regularly come together to share knowledge and experiences, aiming to improve their practice and learn from one another [33]. The exchanges within our community of practice were explicitly shaped and informed by the events and experiences emerging from the twelve participating cases. Hence, we refer to it as a *multiple case-informed community of practice*.

**Figure 1:**
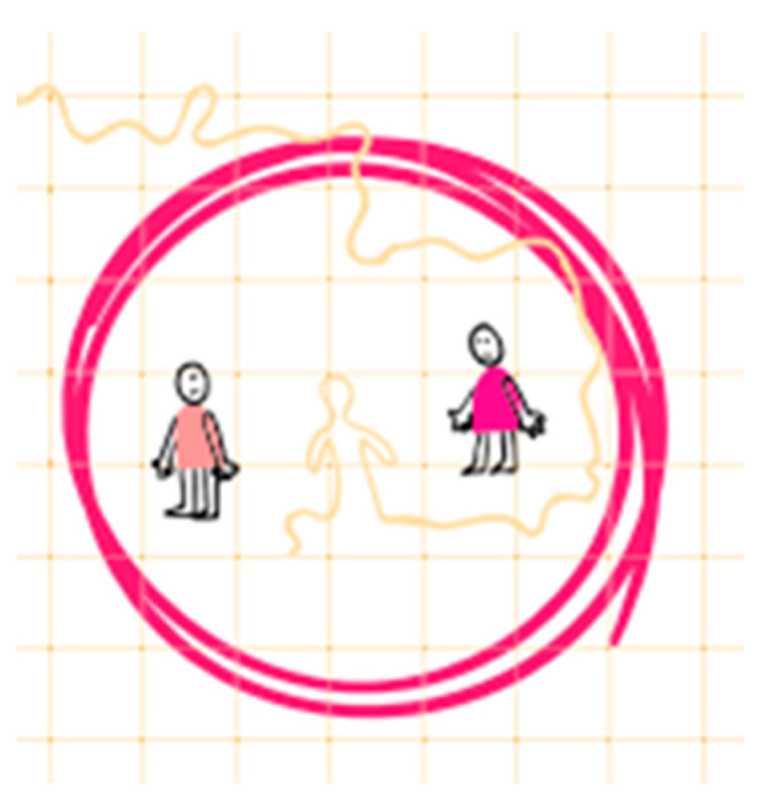
First layer of exchange: case level.

Our community of practice consisted of four layers of exchange. In the first two years (2019–2021), we focused almost exclusively on the first layer, i.e., the case-level around the protagonist (see Figure 1), in order to make sure that the intended process of *getting to(wards) know(ing) together* was grounded in relations with and between people in the stagnant care practices. Hence, in year 1, the outsider-researchers were primarily invited to develop, question, navigate, and communicate their perspectives in and on the everyday routines and interactions between ‘their’ protagonist, insiders, and relatives by means of participant observation and semi-structured interviews. Then, by the end of year 1, each outsider-researcher exchanged and discussed their perspective in a structured meeting with the insiders (and a relative, in two instances). In these case-level exchange gatherings, the insiders (and relatives) were invited collectively to discuss the observations, reflections, and questions of the outsider-researcher, in order to reflect together on the raised issues and exploring alternative responses. On top of that, at the start of year 2, we introduced core teams within each case, aimed to interweave the outsider-researcher’s observations, reflections, questions, and insights—and the resulting interventions—more structurally with the insiders’ expertise and routines. These core teams consisted of the protagonist’s primary staff member [Dutch: persoonlijk begeleider], a relative or second staff member, a manager, a behavioural specialist, the outsider-researcher, and the first author.

**Figure 2:**
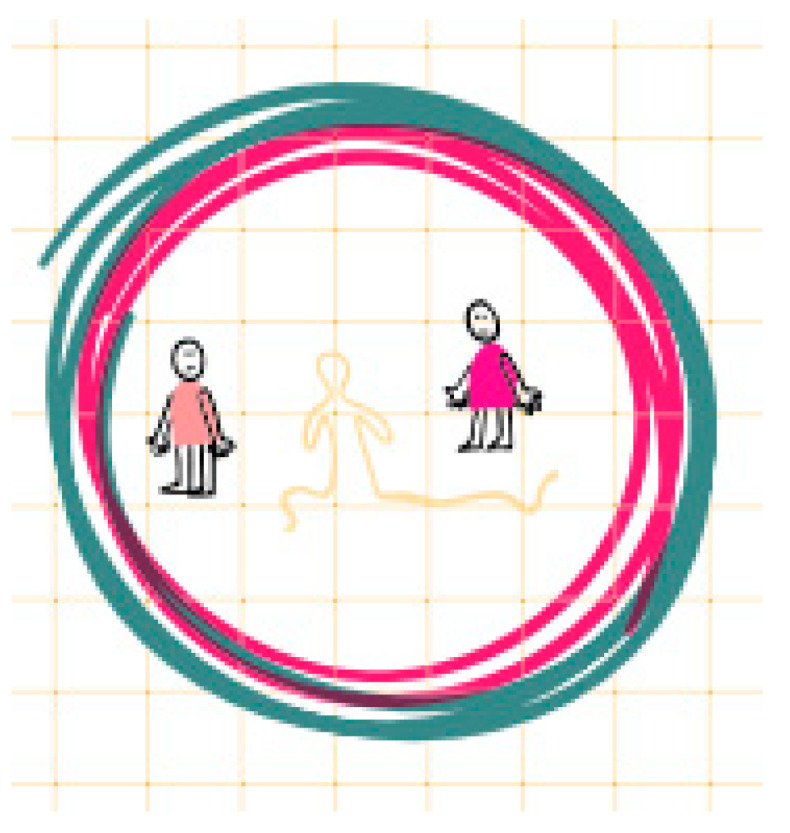
Second layer of exchange: within care organizations.

The second layer of exchange within the community of practice involved the first and second author, who initiated and maintained regular contact with interested staff, other professionals, and executives within each care organisation (see Figure 2). Thus, besides and beyond the case-level focus, over the course of the project (2019–2022) other employees from the care organisations were regularly invited and facilitated to participate in the conversation about, and reflect on, what was being navigated and learned within the cases in their organisation.

**Figure 3:**
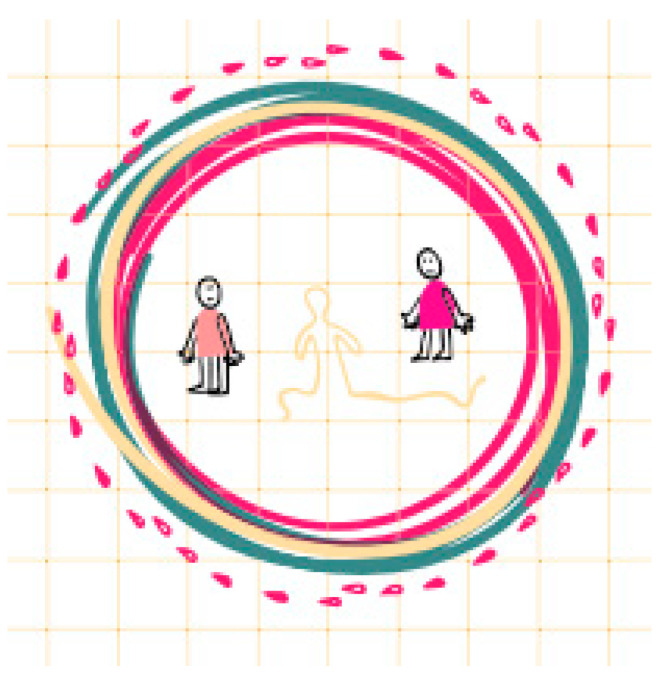
Third layer of exchange: between care organizations.

To facilitate a third layer of exchange, we widened the scope from an *intra*-organisational to an *inter*-organisational level, i.e., between the six partaking care organisations (see Figure 3), by organising five semi-annual meetings—one in year 1, three in year 2, one in year 3. In these broader *outer circle gatherings*, we aimed to explore the many issues that emanated in each case but could not be sufficiently responded to on the case level (e.g., staff turnover, organisational distrust, collaboration challenges, etc.). These inter-organisational meetings were attended by insiders, outsider-researchers, and some relatives from every case, as well as other interested care organisation officials, members from various interest groups, and lecturers and students from applied universities for social work and nursery.

**Figure 4:**
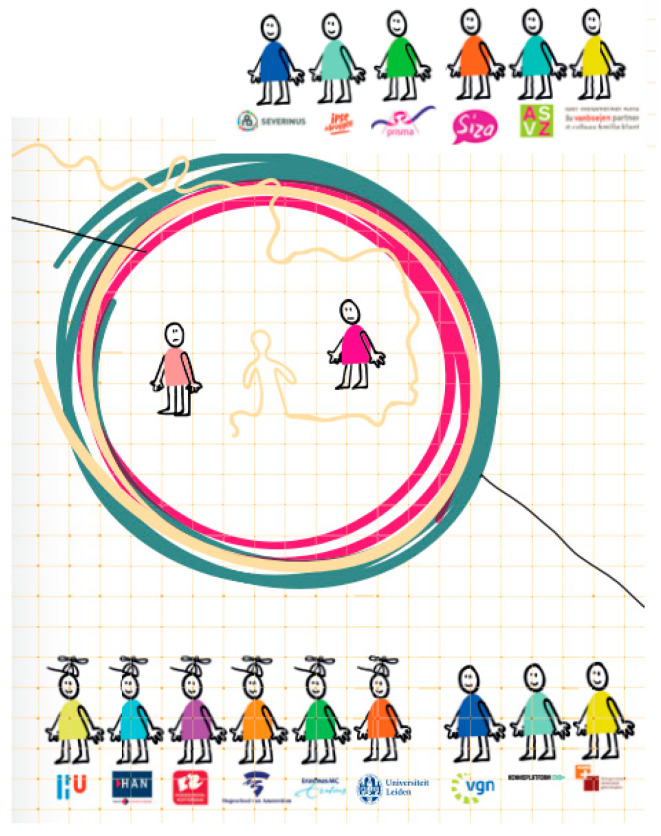
Fourth layer of exchange: between care, educational, and academic contexts.

Lastly, as a fourth layer of exchange, we stimulated active involvement and learning-by-doing within educational and academic contexts (see Figure 4), via the students and registrars from various institutions actively engaged with the events and interactions in the cases. Eleven students from applied universities for social work and nursery as well as two registrars from the training institute for specialized doctors completed internships and graduation assignments. Additionally, groups of students from said applied universities and from the master specialisation Clinical Child and Adolescent Studies attended eight guest lectures, provided by the first and second author, and another senior researcher (K.K.), in which insights and questions from the project were discussed.

### 2.5. Project Aim: Facilitating a Long-Term Exchange Between in- and Outsiders’ Perspectives

By organizing Project WAVE as a multilayered, multiple-case-informed community of practice, with a mixture of prolonged participant observation, interviews and core teams on the case-level, as well as intra- and inter-organisational exchanges and student involvement, we engaged a wide variety of stakeholders, with in- and outsider’s perspectives in a knowledge creation process for over three years. In doing so, we aimed to create space for an unprecedented long-term exchange of experiences, knowledges, and perspectives regarding stagnant care practices around people whose behaviours challenge. Thus interactively developing alternative interpretations amongst people in multiple positions, relationships, and contexts, we wanted to explore their relevance for all involved in practicing and organizing good care.

### 2.6. Positions Within the Academic Research Team

The authors of this article embodied varying roles, capacities, and responsibilities within the academic research team. The first author was principal investigator. He is a university based senior researcher in the tradition of responsive evaluation, and a disability scholar, who specializes in critical-ethnographic, phenomenological, and collaborative research, mostly regarding people with severe intellectual disabilities. He is involved in various long-term projects which aim to create more space for interpersonal exchange and connectedness, in order to increase mutual understanding and the quality of life of everyone involved.

The second author was co-investigator. At the time, she was employed by the Centre for Consultation and Expertise (CCE), where she served as a case coordinator in stagnant care situations and as a science practitioner. She wrote her PhD thesis about the relationships between the organisational environments of residential disability service organisations and challenging behaviours in residents with intellectual disabilities. Furthermore, she contributed to the multidisciplinary guidelines for challenging behaviour in adults with intellectual disabilities and challenging behaviour. She works as primary therapist and advisor to the board of a residential care organization.

The third author was involved as a critical friend. He is a university-based care ethicist and experienced qualitative researcher whose line of research focuses on precarious practices of care and well-being of and for the (chronically) vulnerable. Having both a chronic illness and a young son with Down’s syndrome and epilepsy, he is intrinsically and professionally motivated to carry out research which is aimed at understanding better what it means to live with a chronic disease or disability and what the everyday aspirations of caregivers entail.

## 3. Epistemological Approach

### 3.1. ‘Getting to(wards) Know(ing) Together’

In Project WAVE, we considered that any appropriate and sustainable way to respond to the complexity of stagnant care practices around people whose behaviours challenge, has to acknowledge that everyone involved partakes as a whole person, i.e., with life and limb, background, convictions, strengths and weaknesses, relationships, experiential and formal knowledge, etc. [34,35,36]. Hence, we approached this collaborative research project [26] as a process of ‘getting to(wards) know(ing) together’ [Dutch: ‘interpersoonlijk kennis-maken’] [3]. This dual epistemological notion implies that (i) everyone involved in the research process (including researchers) begins to get to know each other, express themselves, attune to each other, and trust each other, *whilst* (ii) creating knowledge together. Hence, the emerging collaborative knowledge (i.e., the ‘knowing together’) cannot be separated from the people and practices involved, as it is personal, situational, relational and interactional by nature.

The process of *getting to(wards) know(ing) together* in Project WAVE was guided by five interrelated and normative methodological tenets [37] (p. 237):

1. Doing right by complexity and confusion, i.e., acknowledging the non-self-evident, and sometimes unsettling, character of interacting with other participants (verbally and non-verbally), thereby doing justice to the asymmetry, turmoil, and fluidity in learning to know each other [38,39]. In order words, we were very alert to identify and navigate any kind of resistance and conflict.
2. Fundamental personal involvement, i.e., everyone involved was considered as a whole person, not merely on the basis of their (familiar) professional role or responsibility. Involvement from human to human, beyond formal divisions, fosters potential for new (inter)personal insights, developments, and challenges [36,40]. Therefore, we were attentive to everyone’s personal preferences, strengths, and weaknesses, for example by personalizing the research meetings and activities, and also by showing ongoing interest in personal issues and experiences.
3. Experimenting within new contexts, i.e., in order to navigate the space for more fitting and sustainable ways to respond to each other in stagnant care practices, everyone involved was asked to commit to engaging in new ways and unfamiliar contexts [3,41]. That way, we aimed to stimulate learning and acting out of one’s comfort zone.
4. Participants as source, means, and owner, i.e., addressing everyone’s emotions, values, and (professional and experiential) knowledge, in order to acknowledge and enable their contribution and belonging to this process of creating new knowledge [42,43]. Since we aimed for fitting and sustainable change, we strived to make everyone’s impact tangible, also for the sake of transferability (see Section 4.3). Creating particular, recognisable, and versatile multimedia materials (e.g., film, photo, exposition, poems, podcasts, illustrations, booklets) was part of this process. In doing so, we ultimately aimed to facilitate residents as well as their relatives and support staff in being understood, supported, and able to express themselves in more fitting and sustainable ways, beyond the involvement of the outsider-researcher.
5. Slowing down, reflecting, adjusting, and holding on, i.e., everyone involved was regularly invited, in differing formations, to reflect together on the aims of our collaboration, and on the experienced potentials and pitfalls. Thus, we confronted participants with their own and others perspectives, performances, and impact. Thereby, we aimed to inquire and alter familiar patterns, which might entangle rather than untangle a stagnant care practice, also demanding profound and prolonged engagement from everyone involved [44,45].

### 3.2. Ongoing Demands of the Design: Keeping Everyone Receptive to Others’ Perspectives

It was clear from the outset that maintaining sustained involvement from all participants over the full duration of the project could not be taken for granted. After all, prolonged engagement is all but self-evident in stagnant care practices around people whose behaviours challenge, often characterized by all kinds of interpersonal turmoil, friction and conflict [46], and the associated high staff turnover [5]. Additionally, explicitly positioning ‘difference’ as the key element for fitting and sustainable change in those stagnant practices, also carried a risk of collision and conflict. Therefore, throughout the project, a lot of effort has been dedicated to ensure that the (increasing number of) participants stayed involved. In order to facilitate the intended long-lasting and demanding exchange between and with outsider-researchers, insiders, and relatives, the first author, as principal investigator, participated fully in the process of *getting to(wards) know(ing) together*. Therefore, he applied three strategies.

Firstly, in line with a collaborative approach [33,47], he invested in a supportive and responsive relationship with each outsider-researcher. This entailed regularly meeting with them individually (and via email), and consequently raising wondering as well as critical questions [48]. Thus, the first author aimed to elicit any implicit knowledge in the outsider-researchers and to stimulate their ability to reflect on their experiences, perspectives, and input, also in relation to the potentially assimilative perspectives of insiders. When necessary, he also coached and supported the outsider-researchers, as the cases they were assigned to could be unsettling and emotionally demanding for everyone involved. Besides discussing difficult situations, this support could also entail him assisting or acting on behalf of an outsider-researcher, e.g., in meetings with insiders and relatives.

Secondly, the first author kept voicing and explaining the goals, ambitions, and means of the project (see Section 2.4) to everyone involved, as well as towards new audiences, in order to help everyone to stay focused to the main question of this study.

Finally, over the course of the project, the first author invested in strong relations with insiders and relatives within each case. After all, when the planned involvement of the outsider-researchers would end (by the end of year 2), he was primarily responsible for the continuation of *getting to(wards) know(ing) together* within the community of practice in year 3. These strong relations also supported the continuation of the process in year 1 and 2, when turmoil emanated in at least eight cases.

In turn, throughout the project, the first author was supported by senior researcher K.K. as well as by the second author, with whom he shared most formal project management tasks (e.g., content-related organization and chairing of meetings, and reporting to the funding body) and some research activities (e.g., conducting interviews with relatives and insiders, analysis, see Section 4.2). The third author, together with the second author, offered him on-demand peer feedback (e.g., based on his observations in a quarterly outsider-researchers’ meeting that he attended) and advice (especially regarding maintaining a supportive and responsive relationship with each outsider-researcher). On a more structural basis, feedback and advice to the academic research team were also provided by a multidisciplinary advisory group.

## 4. Getting to(wards) Know(ing) Together: Collecting Data

### 4.1. ‘How to’ Getting to(wards) Know(ing) Together: Methodological Components and Phases

Although our community of practice emerged in part as an iterative process, it did contain six core methodological components, i.e., participant observation, reflection, dialogue, imagination, experimentation, and analysis. These intended data collection interactions recurred cyclically throughout the process of *getting to(wards) know(ing) together*, varying in form and intensity across cases and time. Indeed, we adapted this process to varying characteristics and relations among protagonist, insiders, relatives, and outsider-researcher. In hindsight, we can identify nine chronological phases between 2019 and 2022 (see Table 1).

### 4.2. Data and Analysis of Our Approach and Process

Three years of participant observation, one-on-one conversations, reflection sessions, semi-structured interviews, reflexive research team sessions core teams, and inter-organisational meetings (2019–2022) yielded a substantial amount of data. The tangible data consisted of 275 written field reports, 52 audio recordings and 8 video recordings of the outsider-researchers, complemented by 43 reports written by the first author, senior researcher K.K., and research assistant (S.S.), on the various group sessions.

The data analysis was interpretive, reflexive, and dialogical, took place throughout the research process (as Table 1 column 5 shows), and was mainly initiated and conducted by the first and second author, in an ongoing exchange with the third author, outsider-researchers, insiders, relatives, and the research assistant.

First of all, ongoing one-on-one analysis took place in the aforementioned meetings between the first author and the individual outsider-researchers in the first two years of data collection (see Section 2.4).

Secondly, after year 1, we organised six online preliminary imaging sessions in with each outsider-researcher was interviewed by two of their peers, the first author, senior researcher K.K. and the research assistant. These sessions yielded multi-layered preliminary insights into the evolving perspectives of each outsider-researcher on their protagonist, the case and their own positionality.

Thirdly, by the end of the outsider-researchers’ assignment to their case (after year 2), we conducted a multi-perspectivist analysis on the perceived input and impact of each outsider-researcher. In order to do so, each of them was interviewed by two outsider-researchers with a journalism background, who asked them to reflect on how they perceived their own input and impact on the case level. The aim of this analytical step was twofold: (1) To help the outsider-researchers gain a thorough understanding of their own position in, and contribution to, the ongoing process of *getting to(wards) know(ing) together*, and (2) to develop a transferable, credible and distinctive written account of each outsider-researcher’s perspective on their case, and their own input and impact (i.e., outsider-researcher profile). In line with this, each outsider-researcher also developed a tangible final product, which illustrated an important theme within their exchange with the protagonist, insiders and/or relatives (e.g., an animation film, jigsaw puzzle, song, poems, pink building blocks for adults, etc. These products are accessible through the Appendix A, Available online: https://cce.nl/onderzoeken/blik-van-de-buitenstaander-project-wave (accessed on 26 August 2025)).

In an attempt to complement, mirror and challenge these self-perceptions, we also organised a series of four online *outsider on stage*-sessions, wherein each outsider-researcher was subjected to 30 min of listening to three of his/her peers, who discussed what they perceived as their colleague’s input and impact with regards to the protagonist, the insiders, and the project as a whole. After this, the outsider on stage had 5 minutes to reflect and respond. These open gossip-style sessions [Dutch: ‘roddelmethode’] [49] were chaired by the first or second author and the research assistant.

Finally, also by the end of year 2, we asked insiders and relatives how they perceived and evaluated the input and impact of the outsider-researcher. The first author initiated reflective sessions within each core team. In parallel, the first and second authors conducted individual interviews with key insiders (i.e., personal mentors, behavioural specialists and/or team leaders) and relatives within each case (*n* = 36).

The resulting reports and products of all of these analytic sessions were validated through member checking via e-mail. Following validation, the first and second author conducted a series of dialogical analysis sessions, to establish the main lessons learned about our novel approach and process (for the purpose of this paper), Additionally, the first and second author, along with the research assistant (who is also trained in architecture and design), jointly structured and designed the content for a coffee table book and an exhibition, based on the outsider-researchers’ final products, aimed at a broader audience Available online: https://www.projectwave.nl/het-project/opbrengsten/het-boek-/ (accessed on 26 August 2025). For more information about the coffee table book and the traveling exhibition, see the Appendix A. For a more elaborate contextualisation and discussion of these outcomes, including some lessons learned on case level, see Bos et al. [28].

### 4.3. Quality Criteria

In line with the main tenets of *getting to(wards) know(ing) together* (see Section 3), we had selected ‘credibility’, ‘authenticity’, ‘reflexivity’, ‘transferability’, and ‘catalytic authenticity’ as the key quality criteria [50,51].

‘Credibility’ and ‘authenticity’ were pursued via our collaborative research design [29]. Throughout the process, we analysed and discussed observations, insights, and themes in a variety of ways with the outsider-researchers, insiders, and relatives, thereby following procedures such as member check, data- and researcher triangulation, peer review, and joint analyses [52,53].

‘Reflexivity’ was operationalized by engaging in ongoing individual and collective reflective activities regarding our positions, tendencies, emotions, intuitions, beliefs, and motives as outsider-researchers and academic researchers, in relation to the research aims, participants, and findings. We employed a variety of approaches: one-on-one, for instance through walks, phone conversations, shared site visits, and written responses to each other’s observation reports and reflections; and collectively, by centring each meeting of the outsider-researchers around the perspective of one participant, inviting others to respond to it. Throughout, we consistently used the same sequence of reflective questions: (1) What stands out for you? (2) How does that make you feel/want to respond? and (3) What questions does this raise? Thus, we aimed to unpack the experiences of and between outsider-researchers, protagonists, insiders, and relatives, as well as the ways in which we all tried to attach meaning to those experiences [54].

‘Transferability’ of the process findings was enhanced by applying creative and evocative means such as podcasts, poems, paintings, and (documentary) films whilst sharing and discussing said findings. Also, we designed a coffee table book, a traveling exhibition with some of the main findings, twelve in-corporate guests lectures (one per case), and a curriculum for staff, behavioural specialists, and managers in complex care settings.

Furthermore, since we intended to stimulate a longer lasting community of practice around stagnant care practices with behaviour that challenges, yet another important quality criterion was ‘catalytic authenticity’, i.e., that everyone involved felt supported by, and wanted to continue with, the collaborative process we had facilitated [50,51].

## 5. Lessons Learned

Through our collaborative research project, as a multiple case-informed community of practice, we learned five key lessons about both the many requirements and the great potential of structurally creating space for multiple perspectives in research within residential care settings for people whose behaviours challenge. These lessons allude to (1) the perseverance of unease, conflict and loneliness; (2) the effort needed to create a sustainable and inclusive space for different perspectives on different levels; (3) the relevance of experimenting and reflecting at the case level within the context of a community of practice; (4) the importance of reawakening awareness of, and listening to, silent voices within a care team; and (5) the practical value of tangible, creative materials to help protagonists and those involved feel acknowledged.

### 5.1. Persevering Unease, Conflict and Loneliness Together Is Key

Initially, most outsider-researchers tended not to view themselves as researchers, but rather as a ‘research tool’ or means to ‘provide information to the project leaders’, whom they saw as the ones primarily responsible for analysing and interpreting this information. In the first year, most of their energy went to observing, getting acquainted with the insiders and the situation of their protagonist, and trying to understand and improve the situation; which was not an easy task. Over time however—through their long-term involvement with the protagonist and the case, reflective sessions with the principal investigator and fellow outsiders, and exchanges within the community of practice—they increasingly came to see themselves as personally connected to the protagonist, to the insiders, and, to a lesser extent, to the relatives. Each of the twelve outsider-researchers went through similar processes of describing and reflecting on their input, impact, and development during the research project, leading up to the conclusion that enduring and persevering unease, conflict, and loneliness together with the insiders and/or relatives was one of the key elements of their assignment. On the one hand, the outsider-researchers’ observations and input were often perceived as unsettling, yielding negative emotions and responses from insiders, some of whom felt threatened in their position or not recognized for their contribution and expertise. Therefore, a shared perseverance and holding on despite ongoing conflict and resistance were not possible in every case. Whilst all outsider-researchers repeatedly experienced moments of doubt and uncertainty—due to a perceived lack of collaboration, difficulties with the project design or sometimes even because of clear opposition from within the case—eventually it became impossible for two of them to continue. For a more elaborate description and discussion, also regarding risk averse team dynamics, see Bos et al. [28]. On the other hand, the outsider-researchers provided insiders with something extraordinary (e.g., forgotten/alternative/new perspectives, questions and materials), impacting insiders as well as relatives and the situation of the protagonist. Insiders, furthermore, labelled the perseverance of the outsider-researchers as ‘simply necessary’, as ‘you were here in the most impossible time with understaffing and organisational crises’, which highlighted the moral dimension of the outsider-researchers’ involvement, beyond their self-ascribed function as a ‘means’ to introduce difference.

Hence, we learned to define ‘perseverance’ differently – not merely as maintaining a relationship with a person with challenging behaviour through ‘unconditional support’ (as in Triple-C [11]) or ‘unconditional love’ (as in Gentle Teaching [55]), nor as a lifelong commitment such as that of relatives. After all, outsider-researchers’ involvement was not meant to be permanent—although three of them transformed to volunteers after their assignment stopped—this project required ‘only’ two years involvement. Therefore, we came to think about perseverance as a sustained and relational willingness to contribute to the shared aims and desires of the intended collaboration between everyone involved. Perseverance as a team effort thus, not as an individual trait.

### 5.2. Creating Space for Difference Demands a Multilayered Approach

Within the process of *getting to(wards) know(ing) together*, the outsider-researchers and project leader were able to create space for forgotten/new/alternative perspectives, questions and materials by collaborating on four intersecting levels. The first level was investing in a personal relation between the outsider-researchers (and project leader) and the protagonist. Due to this insistent approach, by the end of their assignment, most outsider-researchers ended up as the person longest involved with the protagonist in the case, yielding their perspectives and positions credibility and relevance. Secondly, in this unusual ongoing interpersonal and reflective exchange between and with insiders (caregivers, behavioural specialist, team leader), many participants opened up to each other in unprecedented ways, and learned alternative and sometimes more constructive perspectives on the other, themselves, and their interactions. Thirdly, the initial barriers in the ‘introduction process’ between relatives and outsider-researchers appeared to be indicative of the limited collaboration between relatives and insiders within the stagnant care practice. In most cases, this collaboration was deemed insufficient and challenging by everyone involved. By taking this troubled collaboration as a venture point, and by trying to understand and facilitate it, we aimed to strengthen the precarious bond between these two key participant groups. We did this by recognizing the seemingly similar responsibilities towards the protagonists as well as their inherently different orientations: respectively that of livelong involvement and responsibility, stemming from love and blood band and that of professional, temporary involvement, based on intrinsic and monetary motivations. As a fourth layer, we enabled a series of unusual dialogue sessions between people throughout the care organisations’ hierarchies, about the relevance of procedures and mutual routines, and how everyone involved could apply these—in line with the paper mission and vision of their organisation—to concretely support the people directly involved in the stagnant care practices around our protagonists. For a more elaborate contextualisation and discussion of these processes, see Bos et al. [28].

### 5.3. Exploring and Experimenting Within a Multilayered Community of Practice Offers Added Value

In light of the above, Project WAVE demonstrates the practical value of a multi-layered and collaborative social experiment, with outsider-researchers as embodiment of difference as a relevant reference point. Supported by a ‘connecting’ principal investigator, the ‘strange’ outsider-researchers were able to expose and question many (socialized) facts and tenacious myths which legitimized distancing approaches or restrictive measures towards the protagonists, and to add stimulate thinking and acting in other-then-typical ways. In doing so, we stimulated critical self-awareness and a questioning, open attitude towards alternative perspectives—combined with an extended period of exchanging experiences, ideas and insights, gained by acting upon said alternative perspectives, followed by new actions, etc.

In addition, we connected the arenas of daily complex care, education and academia to each other. Constantly emphasizing how the situations of the protagonists, relatives, and insiders were the basis of learning as a student, and for new scientific insights, encouraged new processes of *getting to(wards) know(ing) together*, in turn enriching the daily care practices.

Through this approach, we refrained from mere theoretical reflection, as well as from attributing blame and responsibility for failures to others, instead stimulating an ongoing praxis of trial and error, i.e., staying explorative into something uncertain together, and supporting and engaging in mutual critique. Thus, ‘experimental-relational space of encounter’ [56] was created between all those involved in our community of practice, in order to make more space for intuitive, (inter)personal knowledge and experience, emotional involvement and moral dilemmas, which tend to be under permanent pressure in strained complex care practices. To foster these tacit and moral sources of knowledge, we firstly focused on ensuring the continuation of the community of practice, especially for the primary staff members. They, in particular, expressed that they appreciated the inspirational and supportive exchange about these topics with colleagues at other locations and institutions—something that they did not have access to in everyday caring practice. Thanks to a government subsidy, it became possible to host this community of practice under the banner of the professional association for primary care workers BPSW for four extra years (2023–2026) Available online: https://www.bpsw.nl/professionals/sociaal-werkers-in-de-gehandicaptenzorg/project-wave-het-vervolg/ (accessed on 26 August 2025). Secondly, we developed a curriculum designed to further facilitate and normalize intercollegiate dialogue and reflection on intuitive, (inter)personal knowledge and experience, emotional involvement, and moral dilemmas in complex care practices Available online: https://www.uvh.nl/onderwijs/ons-aanbod-voor-professionals/voor-professionals-in-zorg-welzijn-en-onderwijs/leergang-jij-en-de-ander-bij-moeilijk-verstaanbaar-gedrag/ (accessed on 26 August 2025). This training module was offered for the first time in the spring of 2025. For more information about the extended community of practice and the curriculum, see the Appendix A.

### 5.4. Attending to Unheard Voices Enables Insight and Change

Every outsider-researcher turned out to prioritize themes in their case that, for various reasons, had received little or no attention in recent years. One the most striking examples of this happened in the case around protagonist Henk, who had received over 35 years of isolating ‘room care’ before participating in Project WAVE. Before being allowed to interact with him, outsider-researcher Mats had to overcome persistent horrifying stories about Henk’s behaviour, which served to legitimate his restrictive daily program and to discourage any spontaneous interaction with Henk. Although said myths were continuously rejected by two team members—residence coordinator René and his colleague Suus—they felt unable to convince their colleagues to change the restrictive program. By gradually getting access to him, Mats overcame his initial perspective of Henk as an ‘inmate’ or ‘a caged tiger’, instead perceiving of him primarily as an old man and a small boy at the same time.

*‘This made Henk both special and very accessible, because we have all been young. I wanted to play with Henk as the little boy he is—a boy who can expend his energy, be mischievous, and explore boundaries. Together with two caregivers, we came up with two games, tested them, and refined them: blocks and a game made of sticks and balls—all in pink, the favourite colour of both Henk and me.’*

(outsider-researcher Mats)

Adopting such a personal, joyous and curious attitude towards Henk helped insiders René and Suus to engage in a series of actions aimed at expanding Henk’s confined living range and social sphere, and slowly entice their colleagues to come along. According to René, the highlight of experimenting with these joyous outings was when he took Henk on a bike ride to the house of Suus, two years after Mats’ introduction.

**Figure 5:**
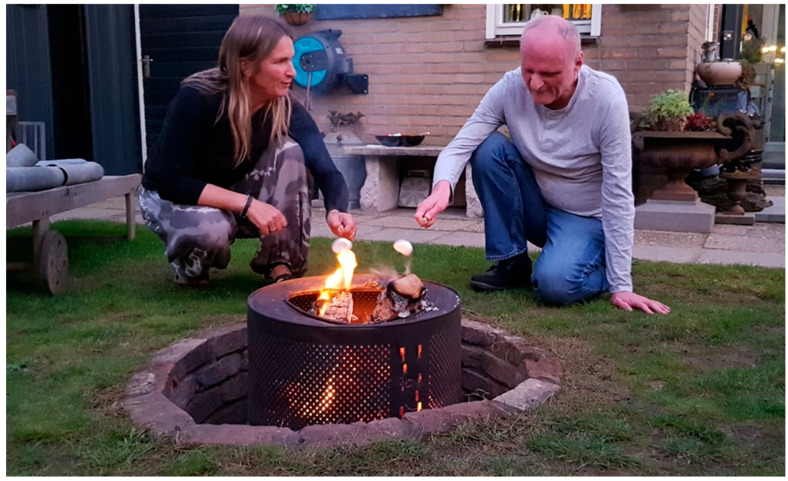
Protagonist Henk and insider Suus in Suus’ garden; photo taken by René.

*‘Last night, I went for coffee with Henk at Suus’s home. We took the tandem bike and arrived there around 7:00 PM. We enjoyed sitting in the gazebo with Suus, and it was wonderful to see how relaxed Henk was and how much he was enjoying himself. Henk also mentioned several times that he felt relaxed and didn’t want to go home yet. Besides having a bonfire, we also roasted marshmallows. A little after 9:00 PM, we headed back home—not because Henk indicated that he wanted to, but because we eventually had to return home. Despite the stories that Henk is afraid of the dark, he actually enjoyed the streetlights, etc. Today, I […] gave Henk the space to process yesterday’s outing; sitting comfortably in his decorated garden, Henk had a lovely time reminiscing. Joi de vivre, long live Project WAVE!’*

(insider René, residence coordinator, email; see Figure 5)

Like Mats in the case around protagonist Henk, each outsider-researcher attended to unheard voices in the case they were assigned to—sometimes from a subordinate subgroup within the team, sometimes from relatives who felt insufficiently acknowledged regarding an issue which was important to them. It is therefore advisable for managers and behavioural specialists in complex care practices to remain aware of the unheard (maybe even: silenced) voices amongst people around a protagonist, to listen to them, and to try to make use of these ‘alternative perspectives’.

### 5.5. Sustainable Multimedia Materials Foster Broader Involvement of Perspectives

Collaboratively creating particular, recognisable, versatile and tangible multimedia materials (e.g., film, photo, exposition, poems, podcasts, illustrations, booklets) was integral part of the process of *getting to(wards) know(ing) together*. We assumed that these collaborative creations would help everyone involved in stagnant complex care practices to relate to each other as whole human beings (rationally, emotionally, embodied, narratively). In doing so, we facilitated our protagonists as well as their relatives and support staff in being understood, supported, and able to express themselves—and contribute to the stagnant care practices in more fitting and sustainable ways, including when the outsider-researcher’s involvement had ended.

An example of those multimedia materials is *Sides to Casper* [Dutch: Kanten van Casper], from outsider-researcher Mark (accessible through the Appendix A. This short documentary film (Available online: https://cce.nl/casussen/casper-en-outsider-onderzoeker-mark (accessed on 26 August 2025)) shows how protagonist Casper appreciated Marks physical proximity and touch—contrary to the prevailing belief among insiders that one-on-one attention, touch, and intimacy with Casper was reserved for relatives and volunteers. Although Casper’s mother and one of the care professionals clearly thought differently, they did not address this in conversations with other insiders, mainly because they feared it would stir friction and alienation. Outsider-researcher Mark however, after listening to their unheard voices, went against the team’s habitus and persistently tried to observe and respond to the subtle—and not so subtle—bodily cues from Casper, all of this much to Casper’s mother’s appreciation.

With this documentary film, Mark initiated several coaching and reflection sessions with the team, about the potential of one-on-one attention, touch, and intimacy. This inspired a change in the team’s habitus: at least three team members started practicing ‘non-functional’ physical one-on-one contact with Casper. One of them, Tonny, reflected on this in a blog Available online: https://www.projectwave.nl/nieuws-en-blogs/tonny-insider/ (accessed on 26 August 2025):

*‘Over the last two years, I did see Mark engaging with Casper, but then I was busy assisting the other residents and all. So it was only after watching the documentary he made about this, that I realised what he had been doing. […] The documentary illustrates what Mark coined as ‘time to-be-together’: a non-functional presence with Casper. After we watched the documentary, me and my colleagues agreed that we would try to embody this ‘time to-be-together’. Upon watching the documentary, to me the everyday meaning of Caspers hands was merely functional, or even negative. Functional, because I primarily hold his hands when I instruct him to do something; negative because Casper frequently uses his hands to grab and pinch whoever is near. Although I often also hold his hand as a sign of my presence during the daily walk, even then my main focus is on preventing behaviour that challenges: the grabbing and pinching of the person who assists him. His hands are always involved in those acts—as are mine, in order to hold him off or to disengage. So, my interpretation and meaning of the relation between his and my hands is strikingly different compared to the intimate image of the hand-in-hand interaction between Mark and Casper*

(see Figure 6)

*It was that image that triggered me the most—and I was eager to try that too with Casper […] As soon as I moved my hand a little bit towards him and the mat, Casper reached to me with his hand. He started to stroke my hand, touch my fingers, fiddle with them. When I replied by imitating what he was doing, he started to grin. […] The first moment Casper touched my hand, I thought to myself: what’s he going to do next? I was very much committed to this being and staying a positive encounter, for both of us. I did not want this touching to turn into pitching; it had to stay good. Casper making positive contact with me with his hands was magnificent. I had not expected it to go so fast—that he would reach out to me like that with his hands, in this setting, and that it would be so pleasant. Sitting beside him without touching would have been fine already. I wanted it to be his decision, since we as staff determine so many things in his everyday life already. I didn’t know that touching hands with him could be positive and non-functional. What I felt in those 4 minutes next to Casper on the doormat? That was good, that was extremely positive. That I, with so little, could have such a successful experience with him, overwhelmed me. It was so easy!*’

(insider Tonny, residential support worker; the complete blog is accessible via the Appendix A)

Thus, the alternative perspective Mark presented—in favour of more one-on-one contact and bodily exchanges between residents and insiders—seemed to fuel change within the team dynamics after it was convincingly shown to insiders in a film based on two years of Marks involvement.

We utilized this and other multimedia materials which had a strong impact at the case level, also to stimulate impact beyond the scope of the project, e.g., in the aforementioned traveling exhibition about Project WAVE, which visited 15 healthcare and educational organizations between 2023 and 2024, as well as in the sessions of the ongoing community of practice for primary staff members and in the curriculum for behavioural specialists and managers that started in the spring of 2025. For a more elaborate contextualisation and discussion of these findings and other examples of multimedia materials, see Bos et al. [28] Available online: https://www.projectwave.nl/het-project/opbrengsten/ (accessed on 26 August 2025). All materials can be accessed via the Appendix A.

## 6. Discussion

Within this unique long-term and multilayered exchange between people with S/M ID whose behaviours challenges, professionals who are trained and socialized within a highly specialized professional care system, relatives, people who gained a variety of knowledges and expertises in other potentially relevant walks of life, and others, we questioned, unsettled, and enriched the logic, routines, and structures of 12 stagnant care practices. By persistently teaming up with the silent (or even silenced) voices of relatives and subordinate team factions, the outsider-researchers were able to create space for hitherto unheard perspectives on good care for the protagonist. Since the exchanges within our community of practice were explicitly shaped and informed by the events and experiences that emerged from sustained engagement at the case level, the aforementioned lessons of our process of *getting to(wards) know(ing) together* may bear relevance for any attempt to establish long-term impact within stagnating complex care settings, both for researchers and for practitioners.

Throughout the project, we learned about (1) the crucial perseverance of outsider-researchers to maintain other yet engaged, (2) the ongoing effort needed to create a structural space for difference on various levels within each care organisation, (3) the importance of trying/exploring within a community of practice, (4) the importance of being aware of and willing to listen to unheard voices amongst care teams and relatives, and (5) fostering the concrete implementation of alternative perspectives through sustainable multimedia materials. Based on these findings, we may conclude that:

(1) in order to facilitate a structural place for alternative perspectives within stagnant complex care practices, building a multilayered community of practice around cases is vital, and
(2) this type of collaborative design is needed to improve the often struggling and instable support services for people with M/S ID whose behaviours challenge.

With regard to harnessing the potential of alternative perspectives on challenging behaviour, particular attention should be paid to the pre-established relationships of key actors in highly specialized residential care settings. Therefore, it is advisable for professionals and teams who support people whose behaviours challenge to consistently engage with the relatives of the protagonist, despite the potential complexities involved. Secondly, it is recommended to professionals to reach out to people within their own social networks who can potentially offer an alternative perspective. Thirdly, it is advisable for managers and behavioural specialists in complex care practices to remain aware of potentially unheard voices surrounding a person whose behaviour challenges, to attend, listen, and to try to make use of these ‘alternative perspectives.’ Fourthly, since the prolonged engagement and contributions of our outsider-researchers were almost everywhere appreciated by insiders, regardless of their specific content, it is advised to explore a less specialized and more interpersonal, relationship-oriented approach to addressing behaviours that challenge—between everyone involved. As persistence and perseverance—despite ongoing turmoil and conflict—proved to be pivotal within out process of *getting to(wards) know(ing) together*, these foundational values may also create a threshold level of stability amongst continuously changing professional care teams.

Consequently, our findings raise questions about the structural support, leadership, and hands-on coaching necessary to sustainably change stagnating situations around residents whose behaviours challenge. Not only embracing a variety of (outsiders’) perspectives within a multilayered learning structure appeared sufficient to trigger change, but the project leader’s close personal involvement and interventions throughout the process of *getting to(wards) know(ing) together* seemed also vital (see Section 3.2). The latter appeared to encourage the majority of participants to stay involved, and to go along with the rationale that any emerging conflict held an opportunity to overcome a barrier towards more (mutual) understanding of the stagnant care practice. Thus, more research is needed on the extend of leadership involvement, as well as on the flexibility and adaptability of individuals and teams in complex care practices, and thereby their ability to assess and do what is necessary to keep from stagnating. In addition, our findings appear to offer starting points for a more active and engaged approach to conducting research within complex care practices—one that allows greater space for interpersonal complexity. At the same time, further research is needed to explore how collaboration and responsibilities among all parties involved in such a layered community of practice can be distributed in an appropriate and sustainable way.

## 7. Conclusions

The central aim of this article was to highlight both what it requires and the great potential it holds to structurally create space for multiple perspectives in research in residential care settings for people whose behaviours challenge. As Clifford Simplican [2] aptly points out, many critiques of current complex care practices tend to oversimplify the systemic and relational complexities and struggles experienced by families and professional networks of someone whose behaviour challenges, primarily focusing on the behaviour of said person. In turn, Clifford Simplican advocates a more nuanced and normative relational position, which aims to improve the quality of life of people with behaviour that challenges as well as their relatives and paid support staff [2]. In her plea for a more fitting—and thus more critical and less reductionist—approach to behaviour that challenges, Clifford Simplican calls for transdisciplinarity and artistic creativity, in order to produce rich and multi-layered imaginaries.

The lessons of Project WAVE add to these suggestions that, in order to engage in fruitful transdisciplinary and creative exchange in complex care practices, a sustainable and supportive learning structure (such as our community of practice, traveling exhibition, and collaborative curriculum) is needed. We recommend that such a layered learning structure explicitly fosters persistent reflection and perseverance to maintain personal relationships between everyone involved; not only people with M/S ID, relatives, and support staff, but also behavioural specialists, managers, directors, outsiders, and researchers. Being open to outsiders’ perspectives in a highly specialized professional care system context is not just a matter of adding creativity and welcoming other disciplines, but also of stimulating widespread personal engagement and mutual trust. Thus, enabling the emergence of interhuman connectedness may help put the often-arduous care practice into yet another perspective, by adding an ongoing sense of belonging despite all differences in positions, perspectives, and privileges.

## Figures and Tables

**Figure 6:**
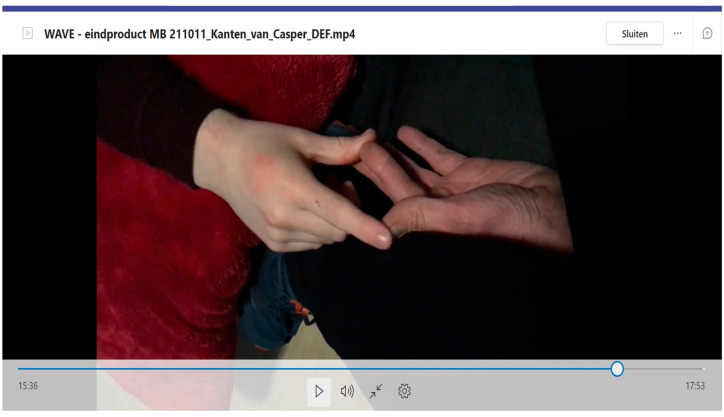
Still from outsider-researcher’s Mark’s documentary film *Sides to Casper*.

**Table 1:** Phasing and methodological components.

Phase	Actual Timeline	Year	Months	Methodological Components
1	December 2019–May 2020	1	1–6	Observing, cautious initial acquaintance (i.e., *participant observation, reflection*)
2	June 2020		7	Initial feedback reflections in exchange meeting per case (i.e., *reflection, dialogue, analysis*)
3	June–November 2020		7–12	Further acquaintance, first outer circle meeting (i.e., *participant observation, reflection, dialogue, analysis*)
4	November 2020–March 2021	2	12–16	Interim imaging, contact from a distance and/or returning inside (due to COVID measures), second outer circle meeting (i.e., *reflection, imagination, dialogue, analysis*)
5	March–July 2021		16–20	Persisting or perishing (turmoil, unease, conflicts, and loneliness); establishing core teams per case; third outer circle meeting (i.e., *dialogue, reflection, analysis*)
6	July–November 2021	3	20–24	Trying out, embodying and embedding new ways of thinking and doing (i.e., *imagination, experimentation, reflection, dialogue, analysis*)
7	November 2021–May 2022		24–30	Reflecting on contributions and impact of outsider-researchers; presentation of final products/insights; fourth outer circle meeting (i.e., *dialogue, reflection, analysis*)
8	May–November 2022		30–36	Farewell as outsider-researchers, organisation-specific meetings; fifth (and final) outer circle meeting (i.e., *dialogue, reflection, analysis*)
9	November 2022–now	>3	beyond 36	Sharing findings via project website, traveling exhibition, learning module and continuation of the community of practice for the next four years (2023–2026) (i.e., *dialogue, reflection, analysis*)

## Data Availability

The data presented in this study are available on request from the corresponding author due to privacy reasons.

## Abbreviations

The following abbreviations are used in this manuscript:

M/S ID	moderate to severe intellectual disabilities
BPSW	Professional association for primary care workers [Dutch: Beroepsvereniging voor professionals in sociaal werk]
CCE	Centre for Consultation and Expertise

## Appendix A

All multimedia materials c.q. tangible final products of the outsider-researchers can be downloaded at https://www.projectwave.nl/links-en-downloads/ (accessed on 26 August 2025). For a direct link to the short documentary film *Sides to Casper* [Dutch: Kanten van Casper] see https://www.projectwave.nl/de-deelnemers/outsider-onderzoekers/mark/. Insider Tonny’s blog is available via https://www.projectwave.nl/nieuws-en-blogs/tonny-insider/ (accessed on 26 August 2025). For more background information, blogs, and audiovisual material see also https://cce.nl/onderzoeken/blik-van-de-buitenstaander-project-wave (accessed on 26 August 2025). For the coffee table book, see https://www.projectwave.nl/het-project/opbrengsten/het-boek-/ (accessed on 26 August 2025). More information about the traveling exhibition is to be found through https://www.projectwave.nl/het-project/opbrengsten/de-expositie (accessed on 26 August 2025). The extended community of practice can be approached via https://www.bpsw.nl/professionals/sociaal-werkers-in-de-gehandicaptenzorg/project-wave-het-vervolg/ (accessed on 26 August 2025). More information about the curriculum is available via https://www.uvh.nl/onderwijs/ons-aanbod-voor-professionals/voor-professionals-in-zorg-welzijn-en-onderwijs/leergang-jij-en-de-ander-bij-moeilijk-verstaanbaar-gedrag/ (accessed on 26 August 2025).
